# Circulating microRNAs in plasma of patients with oesophageal squamous cell carcinoma

**DOI:** 10.1038/bjc.2011.198

**Published:** 2011-06-14

**Authors:** S Komatsu, D Ichikawa, H Takeshita, M Tsujiura, R Morimura, H Nagata, T Kosuga, D Iitaka, H Konishi, A Shiozaki, H Fujiwara, K Okamoto, E Otsuji

**Affiliations:** 1 Division of Digestive Surgery, Department of Surgery, Kyoto Prefectural University of Medicine, 465 Kajii-cho, Kawaramachihirokoji, Kamigyo-ku, Kyoto 602-8566, Japan

**Keywords:** oesophageal cancer, microRNA, plasma, biomarker

## Abstract

**Background:**

Several recent studies demonstrated that microRNAs (miRNAs) are stably detectable in plasma/serum. We hypothesised that plasma miRNAs concentrations contributed to potential biomarkers in patients with oesophageal squamous cell carcinoma (ESCC).

**Methods:**

We selected three oncogenic miRNAs (miR-21, miR-184, miR-221) and one tumour suppressive miRNA (miR-375), which are frequently reported in squamous cell carcinoma, as candidate targets for this plasma miRNA assay. This study was divided into three steps: (1) Determination of appropriate plasma miRNAs in preliminary tests. (2) Evaluation of whether the plasma miRNA assays could monitor tumour dynamics. (3) Validation study on the clinical application of plasma miRNA assays in 50 ESCC patients and 20 healthy volunteers.

**Results:**

(1) In preliminary tests, the plasma level of miR-21 was significantly higher (*P*=0.0218) and that of miR-375 (*P*=0.0052) was significantly lower in ESCC patients than controls. (2) The high plasma miR-21 levels reflected tumour levels in all cases (100%). The plasma level of miR-21 was significantly reduced in postoperative samples (*P*=0.0058). (3) On validation analysis, the plasma level of miR-21 tended to be higher in ESCC patients (*P*=0.0649), while that of miR-375 was significantly lower (*P*<0.0001) and the miR-21/miR-375 ratio was significantly higher (*P*<0.0001) in ESCC patients than in controls. The value of the area under the receiver-operating characteristic curve (AUC) was 0.816 for the miR-21/miR-375 ratio assay. Patients with a high plasma level of miR-21 tended to have greater vascular invasion (*P*=0.1554) and to show a high correlation with recurrence (*P*=0.0164).

**Conclusion:**

Detection of circulating miRNAs might provide new complementary tumour markers for ESCC.

Oesophageal cancer is the eighth most common cancer in the world and is the sixth leading cause of cancer mortality (Enzinger and Mayer, 2003). There are two histologic types of oesophageal cancer, but oesophageal squamous cell carcinoma (ESCC) accounts for ∼90% of oesophageal carcinomas diagnosed in Asian countries (Hiyama *et al*, 2007). Although surgical techniques and perioperative management have progressed, ESCC remains one of the most aggressive carcinomas of the gastrointestinal tract. Therefore, primary tumours must be detected at an early stage, and recurrent disease must be diagnosed when it is still minimal or clinically occult, in order to improve the cure rates for patients with ESCC.

As finding molecular targets for ESCC treatment might help to improve the survival of patients with this lethal disease, studies have attempted to identify the biological factors involved in the malignant potential of ESCC. Several recent studies have elucidated that certain molecules, such as p53, cyclin D1 and FAS, have important roles in tumourigenesis and the development of the ESCC (Hollstein *et al*, 1990; Adélaide *et al*, 1995; Gratas *et al*, 1998). In clinical setting, however, few molecules have been assayed as therapeutic and/or diagnostic biomarkers. Conventional serum tumour markers, such as carcinoembryonic antigen (CEA) and squamous cell carcinoma antigen (SCC), have been used as convenient diagnostic assays (Kosugi *et al*, 2004; Mroczko *et al*, 2008) for early detection and monitoring tumour dynamics of ESCC. These serum tumour markers, however, lack sufficient sensitivity and specificity to facilitate early detection of cancer. Therefore, the significance of detecting novel biomarkers using a less invasive diagnostic assay for ESCC should be emphasised.

MicroRNAs (miRNAs), which are small and non-coding RNAs, regulate the translation of specific protein-coding genes. Since their discovery in 1993 (Lee *et al*, 1993), altered expressions of miRNAs have been associated with several diseases, and tumour miRNAs are involved in tumourigenesis and the development of various cancers. Recent several studies demonstrated that miRNAs are stably detectable in plasma/serum (Calin and Croce, 2006; Chen *et al*, 2008; Filipowicz *et al*, 2008). Tumour-derived miRNAs are resistant to endogenous ribonuclease activity because these may be packaged by some kinds of secretory particles including apoptotic bodies and exosomes in plasma/serum (Hasselmann *et al*, 2001; Mitchell *et al*, 2008; Cocucci *et al*, 2009; Kosaka *et al*, 2010). Therefore, miRNAs can be present in a remarkably stable form (Mitchell *et al*, 2008) and the expression level of serum miRNAs is reproducible and consistent among individuals (Chen *et al*, 2008; Mitchell *et al*, 2008). Furthermore, secretory vesicles including miRNAs can function as intercellular transmitters (Valadi *et al*, 2007; Skog *et al*, 2008; Rechavi *et al*, 2009). Indeed, many studies have demonstrated the presence of circulating miRNAs and their potential use as novel biomarkers of cancers, such as prostate cancer (Mitchell *et al*, 2008), leukaemia (Lawrie *et al*, 2008), oral cancer (Wong *et al*, 2008), pancreatic cancer (Wang *et al*, 2009), colorectal cancer (Ng *et al*, 2009), ovarian cancer( Resnick *et al*, 2009), lung cancer ( Hu *et al*, 2010), breast cancer (Heneghan *et al*, 2010 ) including our report in gastric cancer (Tsujiura *et al*, 2010). These findings should open up a new and interesting field in the screening and monitoring of ESCC patients. However, to date, there has not been any report on the role of circulating miRNAs in plasma of patients with ESCC.

In this study, we selected three oncogenic miRNAs (miR-21, miR-184, miR-221) and one tumour suppressive miRNA (miR-375), which are frequently reported in squamous cell carcinoma, as candidate targets for this plasma miRNA assay. (Chang *et al*, 2008; Feber *et al*, 2008; Guo *et al*, 2008; Wong *et al*, 2008; Avissar *et al*, 2009). We hypothesised that plasma miRNAs concentrations would contribute to potentially useful biomarkers in patients with ESCC. Namely, we investigated whether assay of plasma circulating miRNAs could detect cancers by comparing findings in ESCC patients and healthy volunteer controls. Then, we evaluated whether the concentration of circulating miRNAs in plasma samples could screen cancer patients, monitor tumour dynamics and whether the levels were associated with clinicopathological factors in ESCC patients.

## Patients and methods

### Patients and samples

Between November 2008 and April 2009, for test scale analysis, 20 preoperative plasma samples were collected from consecutive patients with ESCC, who underwent curative esophagectomy (R0 or R1) at Kyoto Prefectural University of Medicine, as well as from 10 healthy volunteers. After the initial quantitative analysis of plasma miRNAs, to validate whether plasma miRNAs are related to primary ESCC lesions, formalin-fixed paraffin-embedded tumour samples were also collected from ESCC patients in order to compare the expressions of miRNAs in primary lesions with those in plasma samples (see the section ‘Relationship between the miRNAs in plasma and primary ESCC tissues’ described below). Furthermore, paired plasma samples were serially collected from eight patients before and 1 month after esophagectomy, showing high miRNAs in plasma in the initial series. For further validation analyses, preoperative plasma samples were collected from another 30 patients with ESCC as well as from another 10 healthy volunteers. Thus, between November 2008 and March 2010, a total of 50 patients with ESCC and 20 healthy volunteers were enroled in this study.

After obtaining informed consent, 7 ml of peripheral blood was obtained from each patient before any surgery and from healthy volunteer controls. Immediately after collection, the blood samples were subjected to isolation of cell-free nucleic acids using a 3-spin protocol (1500 r.p.m. for 30 min, 3000 r.p.m. for 5 min, 4500 r.p.m. for 5 min) to prevent contamination by cellular nucleic acids. Plasma samples were then stored at −80°C until further processing. The resected ESCC specimens were fixed in buffered formalin and embedded in paraffin for pathological examination by standard methods. Macroscopic and microscopic classification of tumours was based on the UICC/TMN staging system (Sobin *et al*, 2009).

### RNA extraction from plasma samples

Total RNA was extracted from 400 *μ*l of plasma using mirVana PARIS Kit (Ambion, Austin, TX, USA), and finally eluted into 100 *μ*l of preheated (95°C) elution solution according to the manufacturer's protocol. Using formalin-fixed paraffin-embedded tissues, total RNA was extracted from four slices of 15 *μ*m thickness (total 60 *μ*m in thickness) using Recover All Total Nucleic Acid Isolation Kit (Ambion), and then eluted into 60 *μ*l of elution solution according to the manufacturer's protocol.

### Protocols for the detection of miRNAs

We selected three candidates for oncogenic miRNAs such as miR-21, miR-184 and miR-221, which were frequently expressed in either ESCC or solid cancers associated with squamous cell carcinoma. Although miR-375 was described as a candidate for tumour-suppressor miRNA in previous reports (Chang *et al*, 2008; Feber *et al*, 2008; Guo *et al*, 2008; Wong *et al*, 2008; Avissar *et al*, 2009), in plasma assay of miRNAs, stable internal controls of plasma miRNAs have not been previously reported. Therefore, we utilised the miR-375 as an internal control in plasma miRNA assay.

The amounts of miRNAs were quantified in duplicate by qRT–PCR using human TaqMan MicroRNA Assay Kits (Applied Biosystems, Foster City, CA, USA). Reverse transcription reaction was carried out with the TaqMan MicroRNA Reverse Transcription Kit (Applied Biosystems) in 15 *μ*l containing 5 *μ*l of RNA extract, 0.15 *μ*l of 100 mM dNTPs, 1 *μ*l of Multiscribe Reverse Transcriptase (50U *μ*l^−1^), 1.5 *μ*l of 10 × reverse transcription buffer, 0.19 *μ*l of RNase inhibitor (20U *μ*l^−1^), 1 *μ*l of gene-specific primer and 4.16 *μ*l of nuclease-free water. For the synthesis of cDNA, the reaction mixtures were incubated at 16°C for 30 min, at 42°C for 30 min, at 85°C for 5 min and then held at 4°C. Then, 1.33 *μ*l of the cDNA solution was amplified using 10 *μ*l of TaqMan 2 × Universal PCR Master Mix with no AmpErase UNG (Applied Biosystems), 1 *μ*l of gene-specific primers/probe and 7.67 *μ*l of nuclease-free water in a final volume of 20 *μ*l. Quantitative PCR was run on a 7300 Real-time PCR system (Applied Biosystems) and the reaction mixtures were incubated at 95°C for 10 min, followed by 40 cycles of 95°C for 15 s and 60°C for 1 min. The cycle threshold (Ct) values were calculated with SDS 1.4 software (Applied Biosystems).

The amounts of plasma miRNAs were calculated on a standard curve constructed using synthetic miRNAs, mirVana miRNA Reference Panel (Ambion). The standard reference miRNAs were amplified for each reaction. However, the expression of miRNAs from tissue samples was normalised using the 2^−ΔΔCT^method relative to U6 small nuclear RNA (RNU6B). The ΔCt was calculated by subtracting the Ct values of RNU6B from the Ct values of the miRNAs of interest. The ΔΔCt was then calculated by subtracting ΔCt of the surrounding normal gastric epithelium from ΔCt of cancer tissues. Fold change in the gene was calculated by the equation 2^−ΔΔCt^ (Livak and Schmittgen, 2001; Pfaffl, 2001).

### Statistical analysis

Mann–Whitney test was used to compare differences in plasma miRNA concentration and miRNA ratio between the cancer group and the healthy group, and Wilcoxon test was used to compare the paired plasma samples obtained before and 1 month after esophagectomy. *P*-value <0.05 was considered significant. *χ*^2^ square test or Fisher's exact probability test was used to evaluate the correlations between the results of the plasma mirRNA concentration and clinicopathological factors. Receiver-operating-characteristic (ROC) curves and the area under the ROC curve (AUC) were used to assess the feasibility of using plasma miRNA as diagnostic tools for detecting ESCC. Younden index was used to determine the cutoff value for the plasma miRNAs concentration (Akobeng, 2007).

## Results

### Evaluation of quantitative RT–PCR for measuring the miRNAs in plasma sample and test scale study to determine the appropriate plasma miRNAs in ESCC

**Figure 1:**
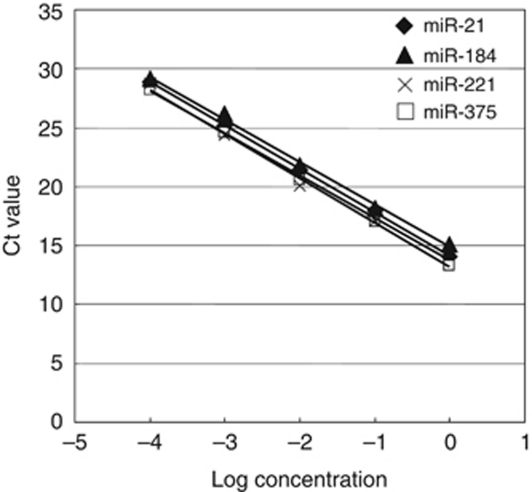
Standard curve of miR-21, miR-184, miR-221 and miR-375 using synthetic microRNAs. Ten-fold serial dilutions of synthetic microRNA were used to generate the standard curves. Linearity was confirmed within these concentrations, ranging from 1 to 0.0001 fmol. (miR-21: *y*=−3.6855x+14.128 (*R*^2^=0.9998), miR-184: *y*=−3.6005*x*+14.877(*R*^2^=0.9960), miR-221: *y*=−3.5932*x*+13.757 (*R*^2^=0.9880), miR-375: *y*=−3.7384*x*+13.242 (*R*^2^=0.9996)).

**Figure 2:**
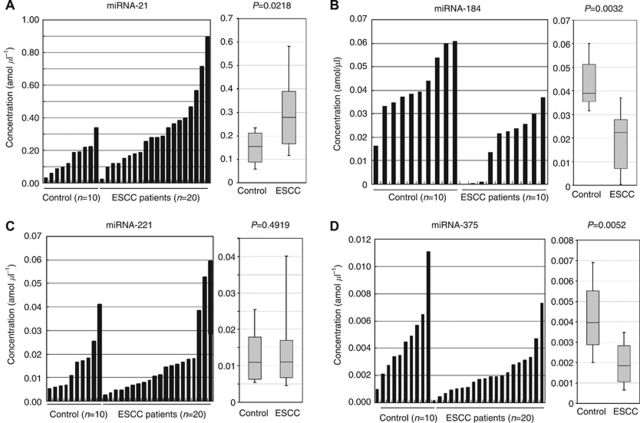
Plasma miRNAs concentration in the initial analysis. Real-time RT–PCR assay, circulating plasma miRNAs such as miR-21, miR-221 and miR-375 excepting miR-184 were detectable and amplified in all samples from 20 ESCC patients and 10 healthy volunteers. Although it was detected in all healthy volunteers, miR-184 could not be detected in half of samples (50%) because its concentration in cancer patients was extremely low. The concentrations of miR-21 tended to be higher in plasma from ESCC patients than in that from healthy controls (*P*=0.0218) (**A**). Whereas, the concentration of miR-221 did not show any significant differences between plasma from ESCC patients and that from healthy volunteers (*P*=0.4919) (**B**). The detectable concentrations of plasma miR-184 in 10 ESCC patients were significantly lower than those in plasma from healthy volunteers (*P*=0.0032) (**C**). The concentrations of miR-375 were significantly lower in plasma from ESCC patients (*P*=0.0052) (**D**). The upper and lower limits of the boxes and the lines inside the boxes indicate the 75th and 25th percentiles and the median, respectively. The upper and lower horizontal bars denote the 90th and 10th percentiles, respectively.

To evaluate the appropriateness of this plasma assay, we first conducted amplification by real-time RT–PCR assay of a 10-fold serial dilution of the mirVana miRNA Reference Panel. The linearity of the quantitative RT–PCR was confirmed from the concentrations of 1 to 0.0001 fmol of each synthetic miRNAs, such as miR-21, miR-184, mir-221 and mir-375 (*R*^2^=0.999, 0.996, 0.988 and 0.999, respectively), between the logarithm of the amount of input miRNAs and the Ct values (Figure 1). Using this assay, circulating miRNAs such as miR-21, mir-221 and mir-375 were detectable in all samples from 20 ESCC patients and 10 healthy volunteers. However, miR-184 could not be detected in half of the samples (50%) because its concentration in cancer patients was extremely low despite being detected in all of the healthy volunteers. However, the concentrations of miR-21 were significantly higher in plasma from ESCC patients than those in healthy volunteers (*P*=0.0218) (Figure 2A). The concentration of mir-221 did not demonstrate any significant differences between plasma from ESCC patients and that from healthy volunteers (*P*=0.4919) (Figure 2B). The detectable concentrations of plasma miR-184 in 10 ESCC patients were significantly lower than those in plasma from healthy volunteers (*P*=0.0032) (Figure 2C). Furthermore, the concentrations of miR-375 were significantly lower in plasma from ESCC patients (*P*=0.0052) (Figure 2D). In oncogenic miRNAs, we hypothesised that plasma miRNAs presented a high level reflecting the tumour miRNA expression in ESCC patients. However, we excluded two miRNAs such as miR-184 and miR-221 for further analyses because these two miRNAs were not sufficiently elevated in the plasma of cancer patients. Therefore, we selected miR-21 and miR-375 as candidates for further analyses.

### Evaluation of whether the concentrations of plasma miRNAs such as miR-21 and miR-375 could monitor tumour dynamics

**Figure 3:**
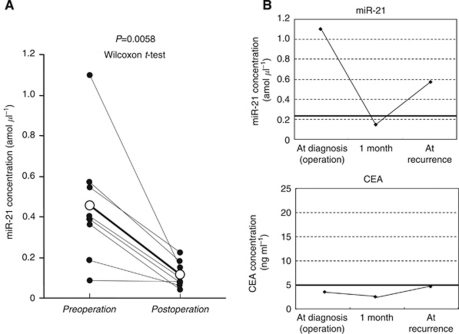
(**A**) Comparison of plasma miR-21 and miR-375 concentrations between pre- and postoperative samples from ESCC patients. The concentration of plasma miR-21 was significantly decreased in the postoperative samples compared with the levels in preoperative samples (*P*=0.0058) (**A**). However, the postoperative plasma miR-375 concentration was not significantly different from the preoperative plasma concentration (*P*=0.2626) (**B**). (**B**) Changes of the plasma miR-21 concentration and the serum CEA concentration in one patient. In one patient, who developed recurrence after surgery without any elevation of conventional serum tumour markers such as CEA, re-elevation of plasma miR-21 concentration was found at recurrence. The boldface lines are cutoff concentrations of miR-21 and CEA.

We analysed the expressions of miRNAs in ESCC tissues and adjacent normal oesophageal tissues in seven ESCC patients showing high concentrations of miR-21 and in four ESCC patients showing low concentrations of miR-21 compared with those in healthy volunteers. Furthermore, seven ESCC patients showing lower concentrations of miR-375 and four ESCC patients showing higher concentrations were analysed. All of the miRNAs obtained from formalin-fixed paraffin-embedded tissues were amplified, and found to be of good quality for amplification (data not shown). Then, we compared the tissue results with plasma results in the miRNA assay. Consequently, miR-21 showed higher expressions in primary ESCC tissues than in normal mucosa in all 11 patients analysed (100%) (Supplementary Table S1), while miR-375 showed lower expressions in primary ESCC tissues than in normal mucosa in all 11 patients (100%)(Supplementary Table S2). Despite a higher expression of tumour miR-21, the plasma miR-21 concentration was low in four ESCC patients (Supplementary Table S1). Similarly, the plasma miR-375 concentration demonstrated normal expression levels despite lower expressions of tumour miR-375 in four ESCC patients (Supplementary Table S2). These findings indicated that the concentration of plasma miRNAs reflected to some extent their expression in tumour. Then, the concentrations of miR-21 were analysed in paired pre- and postoperative plasma samples from eight ESCC patients who underwent curative esophagectomy. The concentrations of plasma miR-21 were significantly reduced in postoperative samples compared with the levels in the preoperative samples (*P*=0.0058) (Figure 3A). In one typical patient, re-elevation of plasma miR-21 concentration was found at recurrence after surgery despite the lack of any elevation in conventional serum tumour marker such as CEA (Figure 3B).

### Validation study on clinical application as a diagnostic plasma biomarker

**Figure 4:**
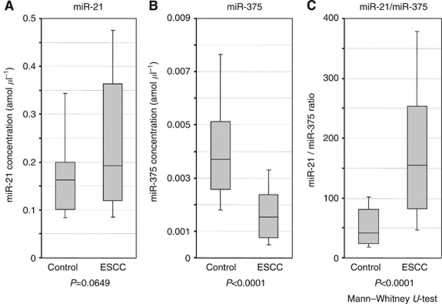
Box plots of the plasma miRNA concentrations in ESCC patients and controls. We analysed another two miRNAs, miR-21 and miR-375, for the validation study. The plasma concentrations of miR-21 tended to be higher in ESCC patients than in controls (*P*=0.0649) (**A**), while the concentration of miR-375 was significantly lower in ESCC patients than in controls (*P*<0.0001) (**B**). The combined biomarker based on the ratio of miR-21/miR-375 was significantly higher in ESCC patients than in controls (*P*<0.0001) (**C**).The upper and lower limits of the boxes and the lines inside the boxes indicate the 75th and 25th percentiles and the median, respectively. The upper and lower horizontal bars denote the 90th and 10th percentiles, respectively.

**Figure 5:**
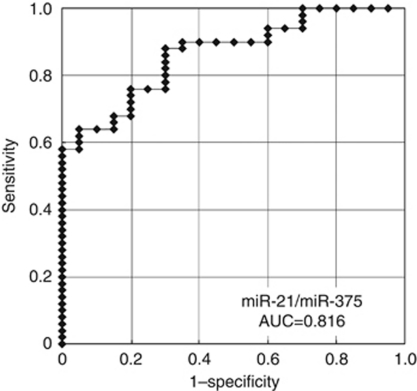
Receiver-operating characteristic (ROC) curve analysis of the miR-21/miR-375 assay ratio for detecting ESCC patients. The ROC analysis showed the greatest AUC of 0.816 for the ratio of miR-21/miR-375.

In total, 50 ESCC patients and 20 healthy volunteers were included in the validation study; there were 15 patients with TNM stage 0–I, 10 with stage II, 24 with stage III and 1 with stage IV. We analysed two miRNAs, miR-21 and miR-375, for the validation study. The plasma concentrations of miR-21 tended to be higher in ESCC patients than in controls (*P*=0.0649) (Figure 4A), while the concentration of miR-375 was significantly lower in ESCC patients than in controls (*P*<0.0001) (Figure 4B). To investigate more sensitive diagnostic biomarkers in plasma, we analysed the ratio of circulating miRNAs levels, dividing the plasma concentrations of miR-21 by that of miR-375, as a combined biomarker. As a result, the ratio of miR-21/miR-375 was significantly higher in ESCC patients than that in controls (*P*<0.0001) (Figure 4C). Supplementary Figure S1 shows ROC curves for both plasma miR-21 and miR-375, and the area under the curve (AUC) were 0.618 and 0.807, respectively. The ROC analysis showed the greatest AUC of 0.816 for the ratio of miR-21/miR-375 (Figure 5).

### Correlation between the results of plasma miRNAs and clinicopathological factors

We examined the association of plasma miRNAs concentrations (miR-21, miR-375) with clinicopathological factors in 50 consecutive ESCC patients. To detect cutoff points that could discriminate cancer patients and normal volunteers using these plasma mirRNA concentrations, we utilised ROC curves with the Younden index (Akobeng, 2007). The cutoff concentrations for miR-21 and miR-375 were 0.2227 and 0.00334 amol *μ*l^−1^, respectively. Consequently, patients with high plasma miR-21 concentration showed a tendency towards vascular invasion (*P*=0.1554) and there was a correlation with disease recurrence (*P*=0.0164) (Table 1). However, there was no significant correlation between the concentration of plasma miR-375 and clinicopathological factors (data not shown).

## Discussion

A non-invasive assay using circulating nucleic acids opens up a new and interesting field in the screening and monitoring of cancer patients. Numerous genetic and epigenetic alterations are known to be involved in tumourigenesis and the progression of various cancer tumours. Several studies have identified tumour-specific alterations in the plasma/serum nucleic acids of cancer patients, and have demonstrated the potential of plasma circulating nucleic acids as new non-invasive biomarkers in patients with various cancers (Sidransky, 1997; Anker *et al*, 2001; Taback and Hoon, 2004; Chan and Lo, 2007; Diehl *et al*, 2008). During the last decade, non-coding RNAs, so-called miRNAs, have also been demonstrated to regulate gene expression by targeting mRNAs for translational repression or cleavage. Consequently, these miRNAs have recently become known as new factors related to oncogenesis and the progression of various cancer tumours (He *et al*, 2005, 2008; Lu *et al*, 2005; Calin and Croce, 2006).

MicroRNAs have been proven to contribute to carcinogenesis and may provide new therapeutic strategies such as biomarkers and therapeutic targets for cancers. Particularly, studies investigating plasma miRNAs comprise an extremely promising field for clinical application. Tumour-derived miRNAs was first described in plasma by Mitchell *et al* (2008). Plasma miRNAs such as miR-141 could efficiently identify prostate cancer patients and show the potential to be new biomarkers. They also demonstrated the high stability of plasma miRNAs after prolonged incubation at room temperature and/or multiple freezing–thawing processes. In addition to this high stability, the characteristics of miRNAs such as tissue-specific miRNA signatures and the availability of many copies per cell would indicate potential advantages as biomarkers compared with those of other nucleic acids, such as circulating DNA and mRNA. In fact, accumulating reports also suggest the potential of miRNAs in the early detection of patients with several malignancies, such as prostate cancer (Mitchell *et al*, 2008), lymphoma (Lawrie *et al*, 2008), oral cancer (Wong *et al*, 2008), pancreatic cancer (Wang *et al*, 2009), colorectal cancer (Ng *et al*, 2009), ovarian cancer (Resnick *et al*, 2009), lung cancer (Hu *et al*, 2010), breast cancer (Heneghan *et al*, 2010) and gastric cancer (Tsujiura *et al*, 2010). To date, however, there has been no report on the role of circulating miRNAs in plasma of patients with oesophageal cancer. These findings prompted us to further investigate the usefulness of miRNAs in patients with oesophageal cancers. In this study, we selected four candidate miRNAs, three oncogenic miRNAs and one tumour-suppressor miRNA, based on their involvement in tumour development and progression of squamous cell carcinoma.

Although some investigators have determined quantities of plasma miRNAs by comparing internal control miRNAs (Ng *et al*, 2009; Resnick *et al*, 2009), it remains controversial as to which miRNAs are suitable as internal control for plasma assay. Therefore, we confirmed a linear correlation between the logarithm of the amount of input synthetic miRNA and the Ct value on real-time PCR, as well as the feasibility of extracting total RNA and amplifying specific miRNA in plasma samples. Based on these findings, we utilised the absolute concentration method for measuring plasma miRNAs in this study. Consequently, as a larger scale study, we selected two candidate miRNAs for plasma screening and monitoring of oesophageal cancer.

Furthermore, we investigated whether plasma oncomir such as miR-21 could be released from primary oesophageal tumours. Comparison between the expression of miR-21 in plasma and primary tumour tissue demonstrated that plasma and primary ESCC tissue samples showed that the high level of plasma miR-21 represented higher expressions in primary ESCC tissues than in normal mucosa in all patients analysed (100%) (Supplementary Table S1). Some patients, however, showed a different pattern of miRNA levels, low plasma miR-21 with a high expression in ESCC tissue. This finding indicated that the expression of plasma miR-21 reflected tumour dynamics to some extent. However, the discrepancies remain to be clarified. One possible explanation for this finding may be owing to the heterogeneity of the primary tumours. However, why tumour miR-21 expression does not influence plasma miR-21 expression in every case remains unclear. We also measured circulating miRNAs in paired plasma obtained before and 1 month after surgical removal of the tumours, in order to confirm tumour release of the circulating miRNAs. As a result, the concentrations of miR-21 were significantly reduced postoperatively in patients with high preoperative plasma miR-21. These findings were similar to those in gastric cancer patients in our previous report (Tsujiura *et al*, 2010). Concerning monitoring cancer, in one representative patient with recurrence, re-elevation of plasma miR-21 concentration was found at recurrence after surgery, although there was no elevation of conventional serum tumour markers such as CEA. These findings also clearly demonstrated that the plasma concentration of miR-21 reflects tumour dynamics and is available as a new plasma biomarker for monitoring cancer. Although the kinetics and metabolism of plasma miRNAs have not yet been clearly elucidated, this issue is currently under evaluation.

To evaluate the possibility for clinical application as novel biomarkers, we performed a large-scale study by increasing the number of plasma samples from ESCC patients and healthy volunteers in order to validate the diagnostic potential of selected miRNAs such as miR-21and miR-375. We found that the plasma concentrations of miR-21 tended to be higher in ESCC patients than in healthy volunteers. However, the concentration of miR-375 was significantly lower in ESCC patients than in healthy volunteers, contrary to our expectation. Previously, we found that a tumour suppressor let-7a showed a lower plasma expression level in gastric cancer patients (Tsujiura *et al*, 2010). We also found that tumour suppressive miR-375 showed a lower plasma expression level in ESCC patients than in healthy volunteers. Surprisingly, comparison between the expression of miR-375 in plasma and primary ESCC tissue samples showed similar tendencies in almost all cases. As circulating miRNAs are considered to have been released from cancer tissues as well as from normal tissues, the majority of these miRNA are expected to have originated from normal tissues. Our findings of some tumour-suppressor miRNAs in cancer patients seemed strange from this perspective. Several recent reports have suggested that plasma miRNAs might be protected in a complex with other molecules, such as exosomes, proteins and lipids (Valadi *et al*, 2007, Mitchell *et al*, 2008). The protection of miRNAs might have greater effect in ESCC patients than healthy volunteers. An alternative hypothesis is that a lower plasma expression of certain tumour-suppressor miRNAs might be due to alterations in miRNAs expressions in normal tissues of cancer patients by unknown mechanisms. Kosaka *et al* (2010) proposed a novel hypothesis that miRNAs could be conducive to the maintenance and surveillance system against cancer progression. During the initial stage of tumourigenesis, downregulation of miRNAs in cancer cells is compensated by the surrounding cells that supply exosomes containing the decreased miRNAs. However, once the surrounding cells can no longer meet this demand, cancer cells enter an advanced stage. Interestingly, this theory would explain our clinical findings. Taken together, the secreted and circulating miRNAs could have a pivotal role as signalling molecules in both physiological events and carcinogenesis. These findings highlight their usefulness as biomarkers and potential therapeutic targets in ESCC.

Determining the expression ratios of genes or miRNAs has been reported to be a useful technique for improving the diagnostic potential of such markers (Gordon *et al*, 2002; Avissar *et al*, 2009). Therefore, we also investigated miRNA expression ratios by dividing the plasma concentration of upregulated miR-21 by that of downregulated miR-375 in order to improve the sensitivity and specificity of plasma miRNA assay for use as diagnostic biomarkers. Then, the ratio of miR-21/miR-375 showed the highest AUC value of 0.816 in the present study, and would be satisfactory for clinical application. The expression ratio of miR-21/miR-375 in plasma could distinguish ESCC patients from healthy control patients with 88% sensitivity and 70% specificity.

We present here a framework to assess tumour characteristics by non-invasive plasma miRNA assay. This study is the first report of circulating miRNAs in plasma of patients with ESCC and our findings demonstrate that cancer-associated miRNAs in plasma can potentially serve as novel non-invasive biomarkers for ESCC. However, many issues should be addressed before these findings can be translated into a clinically useful, non-invasive screening strategy for ESCC. These issues are currently under evaluation in a large number of studies, including other miRNA candidates in ESCC. In conclusion, this study clearly demonstrated that plasma miRNAs such as miR-21 and miR-375 provide useful biomarkers for screening ESCC, monitoring tumour dynamics and predicting associated clinical factors of ESCC. This technology could facilitate clinical decision-making and be applicable to tailor-made medicine for each individual.

## Figures and Tables

**Table 1:** Correlation between plasma miR-21 concentration and clinicopathological factors in consecutive 50 ESCC patients

		**Plasma miR-21 concentration**		
**Variable**	***n***	**High**	**Low**	***P-*value** a
Total	50	24 (48.0%)	26 (52.0%)	
				
*Sex*				
Male	44	21 (47.7%)	23 (52.3%)	0.6688
Female	6	3 (50.0%)	3 (50.0%)	
				
*Age*				
<65	25	12 (48.0%)	13 (52.0%)	1
65⩽	25	12 (48.0%)	13 (52.0%)	
				
*Lymphatic invasion*				
Negative	34	16 (47.1%)	18 (52.9%)	0.9130
Positive	16	8 (50.0%)	8 (50.0%)	
				
*Venous invasion*				
Negative	35	14 (40.0%)	21 (60.0%)	**0.1554**
Positive	15	10 (66.7%)	5 (33.3%)	
				
*T stage* b				
T0/1/2	25	12 (48.0%)	13 (52.0%)	1
T3/4	25	12 (48.0%)	13 (52.0%)	
				
*N Stage* b				
Negative	22	9 (40.9%)	13 (59.1%)	0.5455
Positive	28	15 (53.6%)	13 (46.4%)	
				
*Stage* b				
0–II	25	13 (52.0%)	12 (48.0%)	0.5712
III–IV	25	11 (45.0%)	14 (55.0%)	
				
*Recurrence*				
Negative	39	15 (38.5%)	24 (61.5%)	**0.0164**
Positive	11	9 (81.8%)	2 (18.2%)	

a *P* values are from *χ*^2^ or Fisher’s exact probability test and were considered significant at 0.05.

b TNM classification. Statistically significant or tended values are in boldface type.
