# Supplementary material for: Circulating microRNAs in plasma of patients with oesophageal squamous cell carcinoma
Source: Br J Cancer. 2011 Jun 14;105(1):104–11. doi: 10.1038/bjc.2011.198 (PMC3137413; doi:10.1038/bjc.2011.198)
Supplement: Supplementary Tables S1-S2 and Figure S1 [file bjc2011198x1.ppt]

## Slide 1
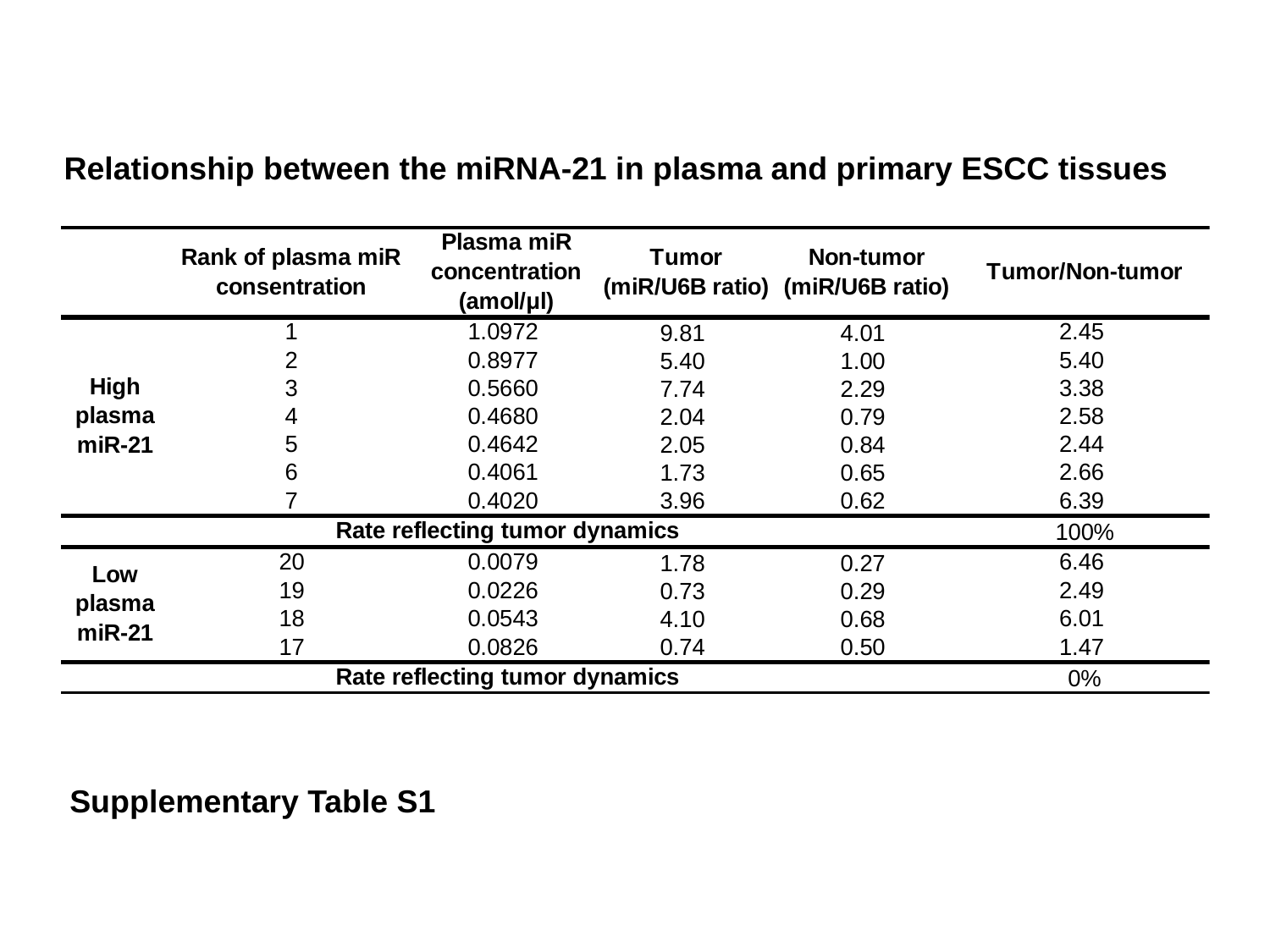

Relationship between the miRNA-21 in plasma and primary ESCC tissues
Supplementary Table S1

## Slide 2
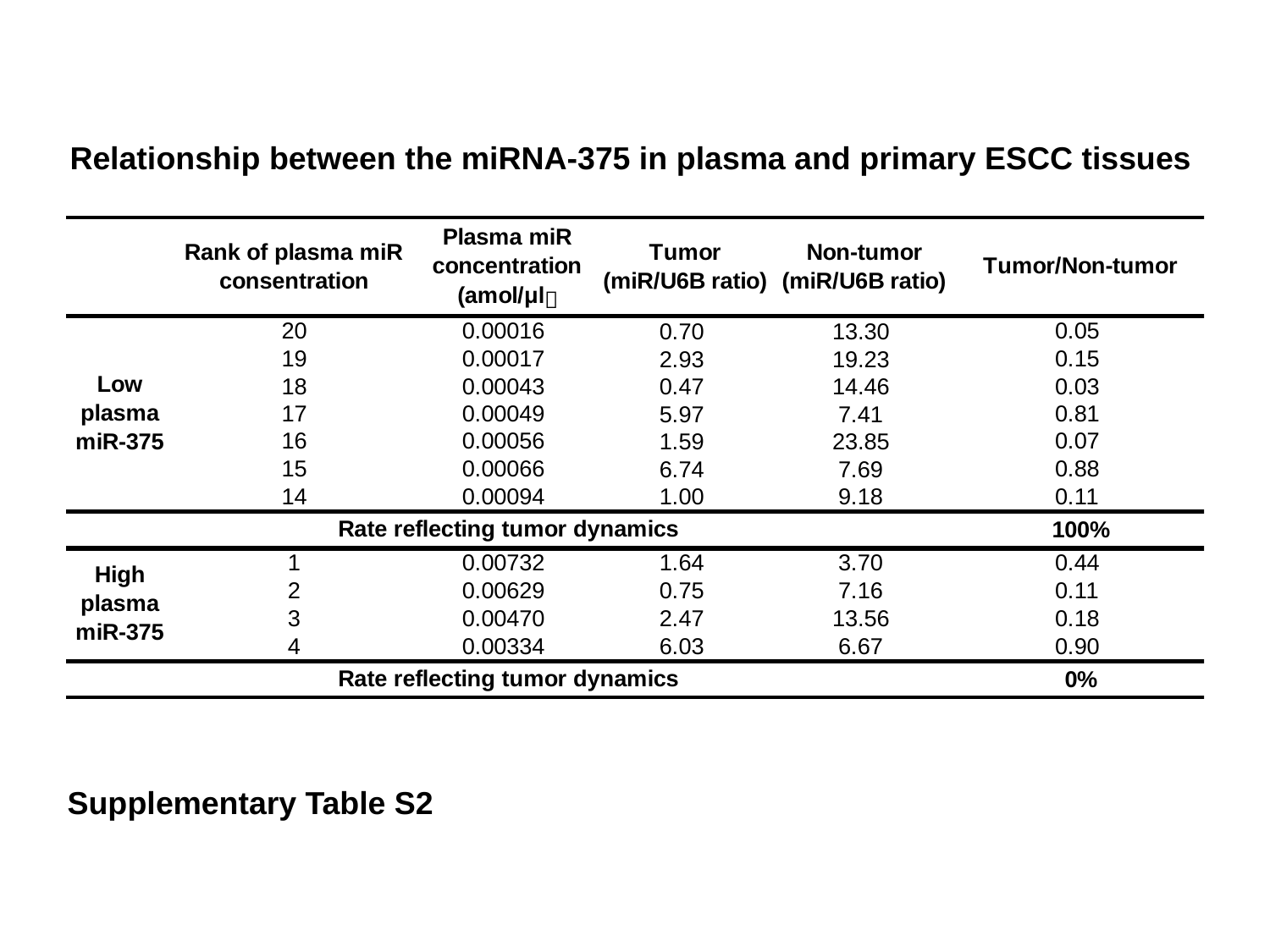

Relationship between the miRNA-375 in plasma and primary ESCC tissues
Supplementary Table S2

## Slide 3
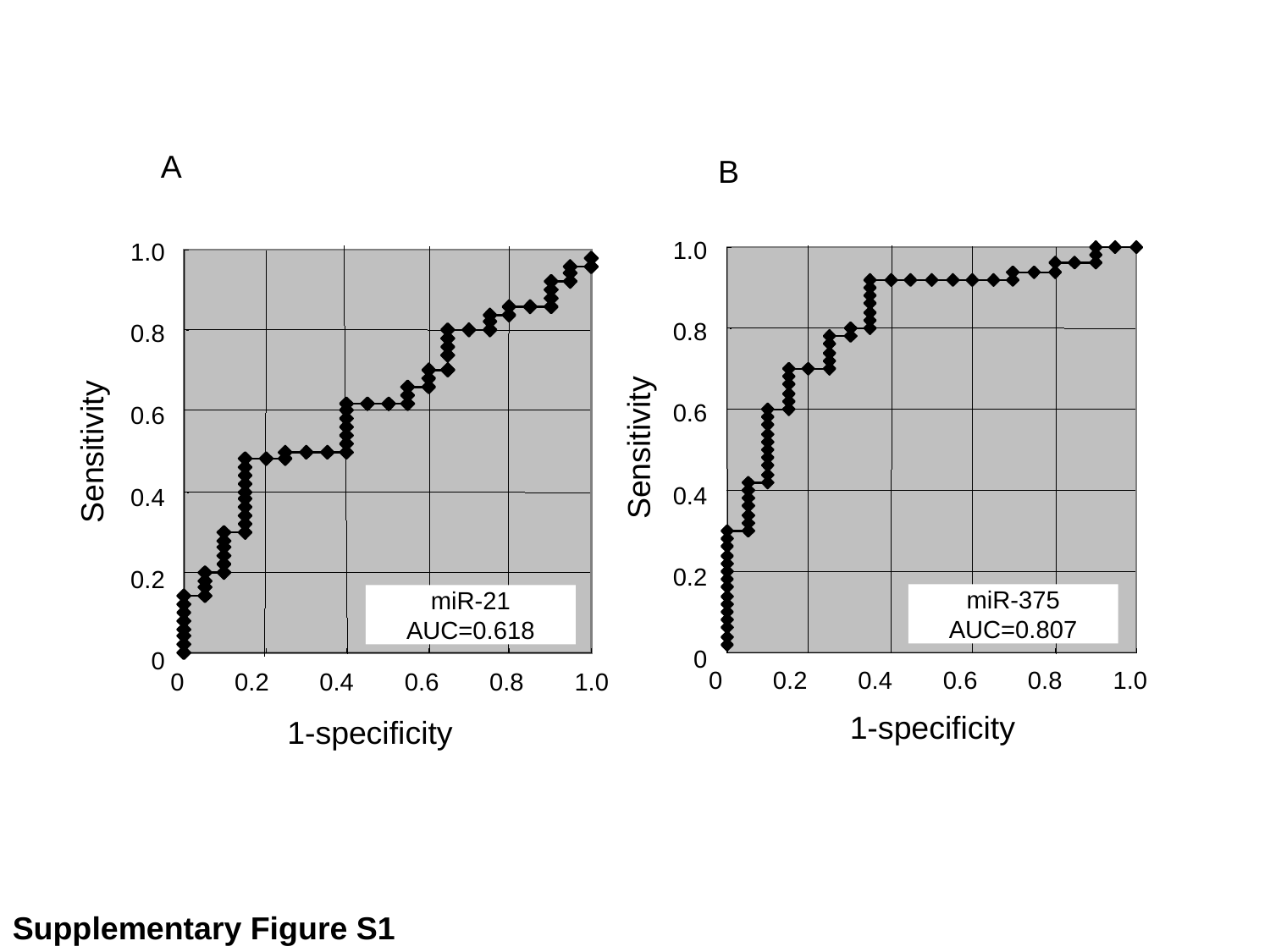

B
A
1.0
0.8
0.6
0.4
0.2
miR-375 AUC=0.807
0
0
0.2
0.4
0.6
0.8
1.0
1.0
0.8
0.6
0.4
0.2
miR-21 AUC=0.618
0
0
0.2
0.4
0.6
0.8
1.0
Sensitivity
Sensitivity
1-specificity
1-specificity
Supplementary Figure S1
