# Supplementary material for: Circulating microRNAs in plasma of patients with oesophageal squamous cell carcinoma
Source: Br J Cancer. 2011 Jun 14;105(1):104–11. doi: 10.1038/bjc.2011.198 (PMC3137413; doi:10.1038/bjc.2011.198)
Supplement: Supplementary Figure Legend [file bjc2011198x2.doc]

**Supplementary Figure legends**

**Supplementary Figure S1**

**Receiver-operating characteristic (ROC) curve analysis in the concentration of both miR-21 and miR-375 assay for detecting ESCC patients**

Concerning the receiver-operating characteristic (ROC) curves for both plasma miR-21 and miR-375, and the area under the curve (AUC) were 0.618 and 0.807, respectively.
